# Identification of SNP and SilicoDArT Markers and Characterization of Their Linked Candidate Genes Associated with Maize Smut Resistance

**DOI:** 10.3390/ijms252111358

**Published:** 2024-10-22

**Authors:** Agnieszka Tomkowiak

**Affiliations:** Department of Genetics and Plant Breeding, Poznań University of Life Sciences, Dojazd 11, 60-631 Poznań, Poland; agnieszka.tomkowiak@up.poznan.pl; Tel.: +48-61-848-7680

**Keywords:** maize, next-generation sequencing, association mapping, maize smut, candidate genes

## Abstract

The implementation of biological advancements in agricultural production is the response to the needs of the agricultural sector in the 21st century, enabling increased production and improved food quality. Biological progress in the maize breeding and seed industries is unique in terms of their social and ecological innovation aspects. It affects agricultural productivity and the adaptation of cultivated maize varieties to market demands and changing climate conditions without compromising the environment. Modern maize resistance breeding relies on a wide range of molecular genetic research techniques. These technologies enable the identification of genomic regions associated with maize smut resistance, which is crucial for characterizing and manipulating these regions. Therefore, the aim of this study was to identify molecular markers (SilicoDArT and SNP) linked to candidate genes responsible for maize smut resistance, utilizing next-generation sequencing, as well as association and physical mapping. By using next-generation sequencing (NGS) and statistical tools, the analyzed maize genotypes were divided into heterotic groups, which enabled the prediction of the hybrid formula in heterosis crosses. In addition, Illumina sequencing identified 60,436 SilicoDArT markers and 32,178 SNP markers (92,614 in total). For association mapping, 32,900 markers (26,234 SilicoDArT and 6666 SNP) meeting the criteria (MAF > 0.25 and the number of missing observations < 10%) were used. Among the selected markers, 61 were highly statistically significant (LOD > 2.3). Among the selected 61 highly statistically significant markers (LOD > 2.3), 10 were significantly associated with plant resistance to maize smut in two locations (Smolice and Kobierzyce). Of the 10 selected markers, 3 SilicoDArT (24016548, 2504588, 4578578) and 3 SNP (4779579, 2467511, 4584208) markers were located within genes. According to literature reports, of these six genes, three (ATAD3, EDM2, and CYP97A3) are characterized proteins that may play a role in the immune response that develops in response to corn smut infection. In the case of genotypes belonging to the same origin groups, markers linked to these genes can be used to select varieties resistant to corn smut. These markers will also be tested on genotypes belonging to other maize origin groups to demonstrate their universality.

## 1. Introduction

Maize (*Zea mays* L.) is considered one of the most important and oldest cultivated plant species. One theory suggests that the most likely ancestor of maize is teosinte (*Zea mays* ssp. *parviglumis*). Genetic loci such as *b1* (teosinte branched 1) and *tga1* (teosinte glume architecture 1) have played a crucial role in the transformation of teosinte into modern maize [1,2,3]. Currently, maize, along with wheat and rice, is among the most economically important cereal crops [4]. The range of maize cultivation is very wide, spanning from 50° N to 40° S. Maize for grain is grown on approximately 197 million hectares worldwide, making it the second most economically important crop after wheat. In comparison, the area under wheat cultivation is 216 million hectares, while rice is grown on 165 million hectares [5]. The annual world maize grain production currently amounts to 1137 million tons, significantly exceeding the production of rice and wheat [5]. Over the past quarter century, maize production has more than doubled, driven by both significant yield increases and expansion into increasingly larger areas [6].

This intensive increase in maize yields would not have been possible without biological advancements. This progress can be described as an ecological method of agricultural intensification, involving the genetic enhancement of plants [7]. The search for new genes of economic significance is a task for modern plant breeding, including resistance breeding.

Currently, the priorities for maize breeding involve obtaining varieties with higher utility value, including increased yield and an improved nutritional, feed, and technological quality of the harvested crop [8]. It is also important to enhance plant resistance to both biotic and abiotic stresses [9]. Biotic stresses involve plant infections by pathogens, including fungal pathogens that cause maize smut. This disease affects all aerial parts of maize [10] and is caused by the fungus *Ustilago maydis*. Spores are the source of fungal infection, which overwinter in or on soil [11,12]. Maize rot occurs almost everywhere maize is grown (e.g., USA, China, Poland). However, the fungus thrives best in dry and warm conditions (between 26 °C and 39 °C). This disease inhibits maize growth and reduces yields, leading to significant economic losses [13]. The most characteristic symptom is the presence of galls. Large galls are typically observed on stems and ears, whereas much smaller tumors are present on leaves and tassels. Chlorosis (coloration changes in plants) may also occur, and additionally, an infected maize ear has altered nutritional value [14]. It contains significantly more protein, particularly higher levels of the amino acid lysine [15]. Unfortunately, such maize is unsuitable for sale because most of the kernels undergo complete degradation. In some countries, smut is used for vaccine production [16]. Resistance breeding is conducted to reduce the incidence of fungal diseases, and it relies on a broad range of molecular genetics techniques, primarily applied in two areas. The first area involves decision-making related to selection based on DNA nucleotide sequence analysis, while the second area focuses on increasing genetic diversity in breeding populations through genetic modifications [17,18]. This not only creates attractive prospects for achieving biological progress but also opens new possibilities for the utilization of not only maize but also other crops [19].

The introduction of molecular tools and rapid advances in next-generation sequencing (NGS) have enabled the sequencing of the genomes of many crop species, including maize. To date, among the most common NGS techniques are 454 pyrosequencing [20], Solexa technology (Illumina, San Diego, CA, USA), SOLiD platform (Life Technologies Corporation, Carlsbad, CA, USA), Polonator (Harvard University, Cambridge, MA, USA), and HeliScope Single Molecule Sequencer (Helicos BioSciences, Cambridge, MA, USA). These technologies provide cost-effective genome-wide sequencing, employing methods such as chromatin immunoprecipitation, mutation mapping, polymorphism detection, and non-coding RNA sequence detection [21]. Sequencing methods such as restriction site-associated DNA (RAD) [22], multiplexed shotgun genotyping (MSG) [23], and bulk segregant RNA sequencing (BSR-Seq) allow for the identification of a large number of markers and a detailed examination of many loci in a small number of samples. The method utilizing the Illumina platform led to the development of genotyping-by-sequencing (GBS) [24] and diversity arrays technology sequencing (DArTseq) [25]. The DArTseq technology was used in the present study to identify candidate genes associated with maize resistance to smut.

The DArT platform offers analyses based on the NGS-DArTseq technology [26,27]. DArTseq analysis generates two datasets: the first dataset contains dominant markers, while the second includes codominant markers with identified single-nucleotide polymorphisms. At least three times as many dominant markers can be obtained using DArTseq compared to the conventional DArT method [28].

These technologies allow for the identification of genomic regions associated with various phenotypic traits, including disease resistance, which is crucial for characterizing and manipulating these regions. The emergence of new genome sequencing technologies, along with novel computational methods, has also led to the sequencing of the maize reference genome. The extensive genotypic data obtained through NGS can be used for association mapping. Genome-wide association studies (GWASs) have thus become a powerful methodology for investigating genetic variation and identifying associations between traits and underlying genetic variability using historical recombination events [29]. Association mapping involves searching for genotype–phenotype correlations in unrelated individuals using dedicated statistical methods [30,31,32]. Association mapping provides the capability to generate high-quality markers for marker-assisted selection (MAS). Functional markers closely associated with a trait reflect gene polymorphisms that directly cause phenotypic variability. Association mapping provides the opportunity to identify specific markers within a broad spectrum of genetic resources. The potential of association mapping arises from the likelihood of achieving higher resolution by utilizing a greater number of recombination events in the history of germplasm development [33].

For several years, maize breeding has been supported globally by useful molecular markers, significantly impacting yield increases not only in the USA but also in other countries. This offers tremendous potential for enhancing the productivity and value of maize germplasm [34,35]. Maize, like barley and rice, is one of the most thoroughly studied cereal species in terms of its genetics. It contains over 32,000 genes on ten chromosomes, with a genome size of 2.3 Gb. A hallmark of the maize genome is its high polymorphism. Many loci have several active alleles, and the frequency of DNA sequence duplications, which include a significant proportion of retrotransposons and transposons, is approximately 58%. Gene-coding regions account for only 7.5% of the entire maize genome [36].

As indicated by the latest literature reports, corn smut in various parts of the world is a current threat and causes a huge decrease in yields. As indicated by research conducted by Ramazanov et al. [37], in Azerbaijan in 2022 and 2023, the infection of corn plants by smut caused a yield loss of 43.19% (2022) and 60.08% (2023). Yield losses were converted into income losses, which amounted to 64.55% in 2022 and 90.99% in 2023. In Hungary, similar losses due to the infection of corn hybrids by smut were recorded by Radocz et al. [38]. Similar studies were published in 2020 in the United States by Muller et al. [39]. They estimated annual corn yield losses caused by diseases in 2016–2019 in 26 states. In their study, the estimated loss per hectare was calculated at USD 138.13. Another study conducted in the Antalya region of Turkey found that the yield loss due to the smut infestation of maize ranged from 20.70% to 45.50% depending on the variety [40]. Also in Turkey, during one study, it was found that due to the smut infestation of maize plantations, a yield loss of 23.10–41.40% was noted depending on the variety [41]. In view of the above, conducting research related to maize resistance to smut seems to be fully justified.

It can be hypothesized that the use of the latest molecular biology techniques to identify genes for smut resistance will reduce the time and costs required to breed new resistant maize varieties. Furthermore, elucidating the role of these genes in the immune response will allow for the development of a sustainable and cost-effective control strategy for *Ustilago maydis* in maize crops.

Therefore, the aim of this study was to identify molecular markers (SilicoDArT and SNP) linked to candidate genes responsible for maize smut resistance, using next-generation sequencing, association mapping, and physical mapping. Identifying specific markers and characterizing the associated candidate genes related to maize resistance to smut will greatly improve the process of breeding new resistant varieties.

## 2. Results

### 2.1. Maize Smut Symptoms

**Figure 1:**
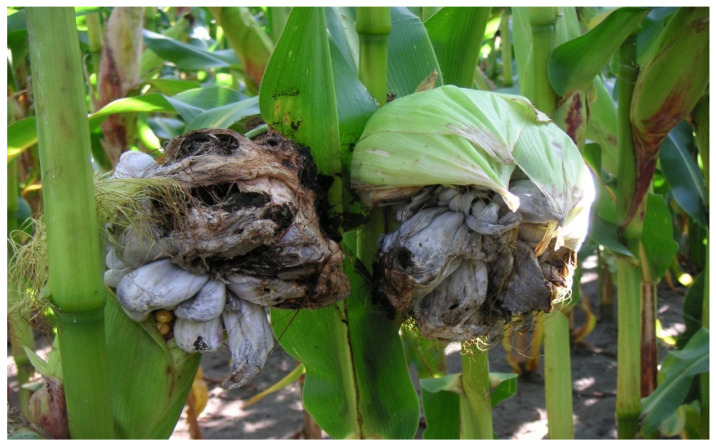
Symptoms of maize smut in Smolice.

In August, symptoms of plant infection by *Ustilago maydis* were observed in experimental fields in Kobierzyce and Smolice (Figure 1). Growths, also known as tumors or galls, appeared on the ears, resulting from the excessive proliferation of infected cells. Initially, the tumors were fleshy and pale, but over time, they turned brown, wrinkled, and cracked. When the galls burst, they spread teliospores on the ground or the plants. In the form of spores, the fungus overwinters on crop residues left after maize harvest. In spring, the fungus begin to germinate and produce 4-cell haploid basidia. Each basidium cell will produce one basidiospore (sporidium). As a result of plasmogamy, when two sexually different sporidia or hyphae growing from their cells come into contact, a dikaryotic cell will be formed. Its hyphae can cause another local infection, and then in the tumors, they will disintegrate into binucleate, spherical teliospores. The period from infection to the production of a new generation of teliospores lasts about 2 weeks.

### 2.2. Phenotyping—Analysis of Degree of Infection of Maize Plants by Smut

Detailed information on phenotypes such as the mean, skewness, kurtosis, standard error, and coefficient of variation observed in each locality, as well as broad-sense heritability, is presented in Table 1. Maize smut infection was significantly higher in Kobierzyce compared to Smolice (Table 2). The large proportion of completely resistant plants in the experiment conducted in Smolice resulted in higher skewness, kurtosis, and coefficients of variation than those observed in Kobierzyce. The broad-sense heritability for maize smut estimated from environments was 59.7 (Table 2).

**Figure 2:**
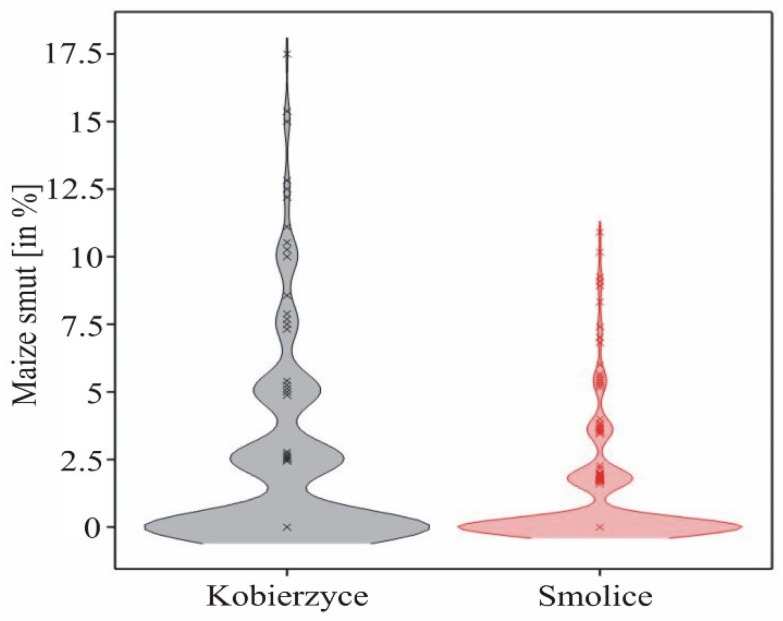
Density charts illustrating maize smut distribution.

The observed maize smut had a normal distribution at both localities, allowing for association mapping. An analysis of variance indicated that the main effects of genotype and location, as well as the genotype × location interaction, were significant for maize smut (Table 3, Figure 2).

Maize smut values ranged from 0% (for 28 genotypes) to 9.34% (for the G02.15 genotype) in Kobierzyce and from 0.00% (for 48 genotypes) to 7.273% (G02.15) in Smolice. On average, for both sites, maize smut values ranged from 0% (for 15 genotypes) to 8.307% (for the G02.15 genotype) (Table 4). The variability in the observed values, expressed in standard deviations, differed between locations, ranging from 0% to 8.036% (G04.03) in Kobierzyce and from 0% to 4.725% (G06.16) in Smolice (Table 4).

### 2.3. Genotyping—Identification of Molecular Markers Linked to Genes Determining Maize Resistance to Smut

Illumina sequencing identified a total of 92,614 markers, including 60,436 SilicoDArT markers and 32,178 SNP markers. For association mapping, 32,900 markers were used, comprising 26,234 SilicoDArT markers and 6666 SNP markers, meeting the criteria of minor allele frequency (MAF) > 0.25 and fewer than 10% missing observations.

**Figure 3:**
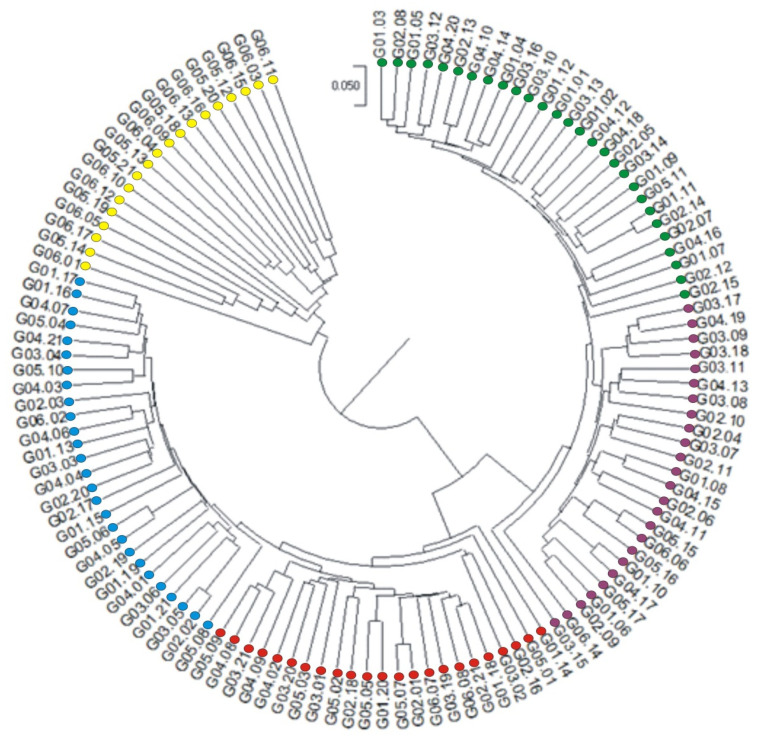
A dendrogram showing the genetic similarity between the 122 hybrids constructed based on 32,900 markers (26,234 SilicoDArT and 6666 SNP).

The identified markers were used to prepare a dendrogram illustrating the genetic similarity between the analyzed hybrids (Figure 3). The dendrogram shows five groups of genetic similarity. Group I and V are particularly noteworthy because they are the most consistent with the origin of the hybrids. The first group includes hybrids from G06.11 to G06.01 (group marked in yellow). This group includes 19 hybrids that come the United States. Group V includes hybrids G02.15 to G01.03 (group marked in green). Of the 28 hybrids 21come from Europe. Group II consists of hybrids from numbers G01.17 to G05.08 (group marked in blue), of which 15 come from Europe and 12 from the USA. Group III contains 23 hybrids from G05.09 to G01.14 (group marked in red), of which 12 are from Europe and 11 from the USA. Group IV comprises 25 hybrids, with 15 originating from Europe and 11 from the USA. In the dendrogram, this group starts from hybrid number C03.15 to hybrid number G03.17 (group marked in purple) (Figure 3). The presence of hybrids from both the USA and Europe in the same genetic similarity groups indicates seed material exchange between different countries and continents. Therefore, when predicting the formula for a heterosis hybrid, it is crucial to consider the genetic similarity, determined based on effective molecular markers, e.g., SNP, be-tween the parental components rather than their geographical origin.

**Figure 4:**
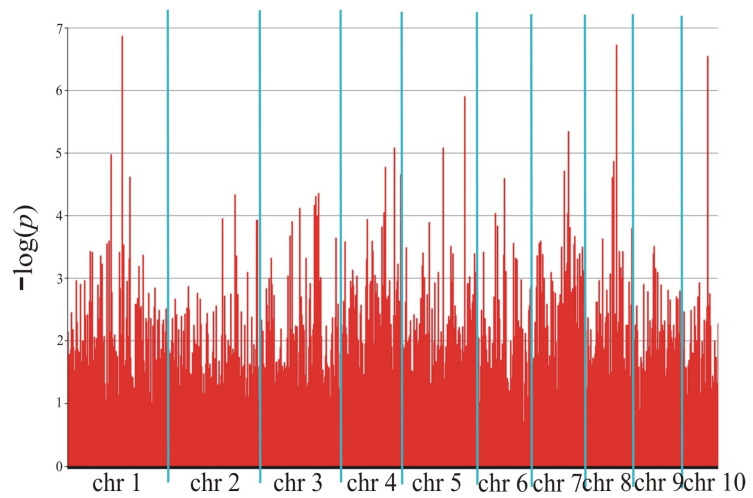
Manhattan plot representing maize smut infection in Smolice.

**Figure 5:**
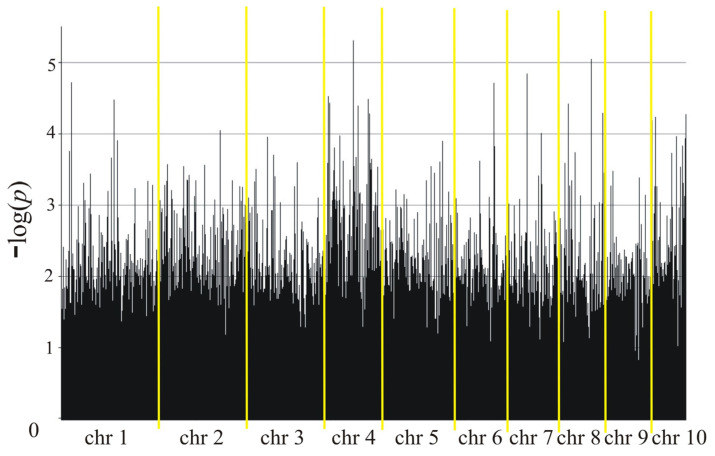
Manhattan plot representing maize smut infection in Kobierzyce.

The genotyping and phenotyping results were used for association mapping, identifying 2609 markers (2136 SilicoDArT and 473 SNP) in Smolice (Table 5 and Figure 4) and 7331 markers (5775 SilicoDArT and 1556 SNP) in Kobierzyce (Table 5 and Figure 5) at a significance level of 0.05. The percentage of variation explained by each marker ranged from 2.4% to 15.4% in Smolice and from 2.4% to 12.0% in Kobierzyce (Table 5). Among the selected markers, 61 were highly statistically significant (LOD > 2.3) (Table 6). The percentage of variation explained by highly significant markers in both localities ranged from 5.6% for SilicoDArT marker 2426908 to 20.1% for SilicoDArT marker 4775057 in Smolice and from 5.6% for SilicoDArT marker 29619676 to 15.3% for SilicoDArT marker 24016584 in Kobierzyce (Table 6).

Among the 61 markers identified as highly statistically significant (LOD > 2.3), 10 (highlighted in color in the Table 6) were significantly associated with plant resistance to maize smut in both locations (Smolice and Kobierzyce) (Table 6). Using the BLAST database, the location of these markers was determined, and the associated candidate genes are listed in Table 7.

In the next step, 10 molecular markers highly significant in both locations (Smolice and Kobierzyce) were localized using the basic local alignment search tool (BLAST), NCBI, and Maize GDB. Table 7 lists their locations and the associated candidate genes. Of the 10 identified markers, 3 SilicoDArT (24016548, 2504588, 4578578) and 3 SNP (4779579, 2467511, 4584208) markers were localized within genes (markers highlighted in color in the Table 7). Among these six genes, three were characterized as proteins that might play a role in the immune response of maize to smut infection. The first gene was the ATPase family AAA domain-containing 3 (ATAD3) protein, which encoded unique mitochondrial proteins. Plants with disrupted ATAD3 showed reduced growth, aberrant mitochondrial morphology, diffuse nucleoids, and a reduced oxidative phosphorylation complex.

The SilicoDArT marker 24016584 was located within this gene. Another important gene was enhanced downy mildew 2 (EDM2), within which SNP 4779579 was located (Table 7). The predicted protein showed typical features of transcriptional regulators. EDM2 contains two putative bipartite nuclear localization signals (NLSs), two zinc-finger-like motifs, a proline-rich region, and a large aspartic acid-rich region. Both zinc-finger-like regions resemble the PHD (plant homeodomain) finger motif. Mutations in EDM2 are associated with RPP7-mediated resistance against *Hyaloperonospora parasitica* isolate Hiks1 (HpHiks1), suggesting that EDM2 may function as a direct or indirect regulator of RPP7 expression. The third important gene was lutein deficient 5, chloroplastic, within which SNP 2467511 was localized. In plants, lutein is present both in chloroplasts and chromoplasts. In chloroplasts, it is a component of the light-dependent phase of photosynthesis; therefore, a reduced amount of lutein negatively impacts this process.

**Figure 6:**
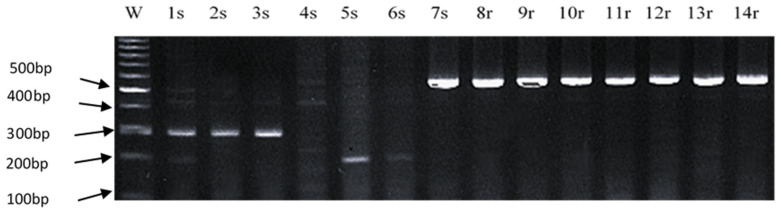
An electropherogram showing a 559 bp product specific to SNP 4779579 and its associated gene—enhanced downy mildew 2. s—susceptible. r—resistant.

Using the polymerase chain reaction (PCR), the identified markers were tested on seven susceptible and seven resistant maize smut genotypes. The primers designed for the identification of the selected markers are listed in Table 8. Among the ten markers, only one SNP (4779579) differentiated between resistant and susceptible maize smut genotypes (marker highlighted in color in Table 8). Numbers 1 through 7 indicate susceptible genotypes, while numbers 8 through 14 indicate resistant ones (Figure 6). A specific product of 559 bp, characteristic of SNP 4779579 and its associated gene, enhanced downy mildew 2, was present in one susceptible genotype (7s) and in all resistant genotypes (8r–14r) (Figure 6). These results suggest that the enhanced downy mildew 2 gene can be associated with maize resistance to smut.

## 3. Discussion

Since the mid-1990s, many research centers worldwide have been conducting intensive studies on the structure and function of the maize genome using advanced biotechnological and molecular biology techniques. As a result of extensive breeding experiments, phenotypic observations, and genetic analyses, many quantitative trait loci (QTLs) associated with specific quantitative traits such as yield and resistance to abiotic and biotic stresses have been identified. The priority for all breeders is to obtain high-yielding and disease-resistant maize varieties [42]. The present study analyzed maize genotypes, both phenotypically and genotypically, to identify molecular markers linked to candidate genes responsible for maize smut resistance. This disease is caused by the fungus *Ustilago maydis*. It is a plant pathogenic fungus that causes tumors on all aerial parts of its host, maize (*Zea mays*). The formation of these prominent symptoms is associated with a comprehensive reprogramming of the host’s physiology, cell morphology, and organ development [42]. An important characteristic of *U. maydis* relevant for its development as a model system in fungal cell biology lies in its bi-phasic life cycle. The fungus initially grows as a saprophytic haploid yeast. Upon encountering an appropriate host surface, the perception of a compatible pheromone signal induces filament formation, leading to the fusion of two compatible cells [43]. The resulting dikaryon represents the pathogenic stage of *U. maydis* and grows strictly in a filamentous form [44]. The ability to induce filamentation and penetration structures (appressoria) in vitro was instrumental for establishing *U. maydis* as a model system in fungal cell biology [45]. *U. maydis* was among the first plant fungal pathogens whose genome was sequenced.

In this study, we focused on phenotypic observations concerning the extent of *U. maydis* infection in 122 maize genotypes that causes smut. Observations were conducted in two locations: Smolice and Kobierzyce. Maize smut infection was observed significantly more in Kobierzyce than in Smolice. The large number of completely resistant plants in the experiment conducted in Smolice resulted in higher skewness, kurtosis, and coefficients of variation than in the experiment conducted in Kobierzyce. The broad-sense heritability for maize smut estimated across environments was 59.7. Higher smut infection rates in Kobierzyce were influenced, among other factors, by environmental conditions, as this location had higher temperatures and precipitation compared to Smolice. An analysis of variance indicated that the main effects of genotype and location, as well as the genotype × location interaction, were significant for maize smut. According to Juroszek and von Tiedemann [46], high temperatures and high humidity increased maize infection by *Ustilago maydis*.

Due to genotype–environment interactions, phenotypic analysis alone may be insufficient to identify maize genotypes resistant to smut. Soto et al. [47] argued that in the era of technological advancement, traditional methods used in breeding are insufficient. In response to this challenge, contemporary breeding programs employ high-throughput plant genome analysis techniques to improve new varieties, including maize [48]. This genomics-oriented approach provides information about coding regions, which reveal details about the structure of a protein (gene), as well as intergenic regions [49]. With advancements in high-throughput DNA sequencing methods, which enable the sequencing of entire genomes and transcriptomes, a new level of research quality has emerged for many plant species, including maize [50,51]. The introduction of next-generation sequencing (NGS) methods has enabled the discovery of nucleotide sequences in plants other than model organisms with small genomes, such as *Arabidopsis thaliana*. In recent years, many researchers have attempted to identify molecular markers functionally associated with important traits in maize. Bocianowski et al. [52] used NGS technology and association mapping to identify markers associated with the heterosis effect in maize. Using the same methods, Sobiech et al. [53] identified markers associated with maize plant resistance to *Fusarium*.

In our research, out of the selected 61 markers, 10 were highly statistically significant (LOD > 2.3) and showed a significant association with plant resistance to maize smut in two locations (Smolice and Kobierzyce). Using the BLAST database, the location of the selected markers was determined, and the associated candidate genes were provided. Among the 10 identified markers, 3 SilicoDArT (24016548, 2504588, 4578578) and 3 SNP (4779579, 2467511, 4584208) markers were localized within genes. Among these six genes, three were well-characterized proteins that might play a role in the resistance response to maize smut infection: 1. ATPase family AAA domain-containing 3 (ATAD3) proteins; 2. enhanced downy mildew 2 (EDM2); and 3. lutein deficient 5, chloroplastic (CYP97A3). Additionally, SNP 4779579, linked to the enhanced downy mildew 2 gene, distinguished between resistant and susceptible genotypes. A characteristic product of 559 bp was present in all maize smut-resistant genotypes under field conditions.

According to Gordon [54], ATAD3 (ATPase family AAA domain-containing protein 3) proteins are newly discovered mitochondrial membrane proteins in *Arabidopsis thaliana*. Studies in metazoans have indicated that ATAD3A localizes to mitochondria–ER contact sites and is involved in a variety of processes required for proper mitochondrial function. However, the role of ATAD3A proteins in plants is less well defined. ATAD3 proteins in *A. thaliana* underwent two gene duplication events, resulting in two clades, both of which are required for plant viability. Research conducted by Zelman [55] indicated that the activity of ATPase family AAA domain-containing protein 3 is linked to plant responses to abiotic stress. Minsoo [56] identified three homologous ATAD3 proteins, involved in mitochondrial nucleoid organization, as interacting with suppressor hot1-4 1 (SHOT1). Importantly, disrupting ATAD3 function leads to impaired nucleoids, a decreased accumulation of complex I, and improved heat tolerance. These proteins increase plant resistance to abiotic stresses, such as high temperature and stress, and may also play a significant role in regulating the plant immune response to biotic stress.

Regarding the second gene significantly linked to maize smut resistance, EDM2, McDowell [57] highlighted its multifaceted role beyond the immune response. The EDM2 mutation also causes pleiotropic effects, influencing flowering time and leaf cellular development, indicating a broad regulatory function [58,59]. The first clues to the molecular function of EDM2 were provided by its protein sequence, which contained a nuclear localization signal, a methyltransferase domain, and plant homeodomain (PHD) fingers associated with epigenetic regulation [60]. Further evidence of an epigenetic role emerged from a yeast two-hybrid screen, which identified interactions between EDM2 and a small family of chromatin remodeling factors [61].

The third important gene associated with maize smut resistance is lutein deficient 5, chloroplastic (CYP97A3). As reported in a publication by Niu et al. [62], this gene is one of the cytochrome P450 enzymes. The CYP97A3 gene, together with the CYP97C1 gene, catalyzes hydroxylations of the β- and ε-rings of α-carotene to produce lutein. Lutein, a dihydroxy derivative of alpha-carotene (beta, epsilon-carotene), is the most abundant carotenoid in photosynthetic plant tissues where it plays important roles in light-harvesting complex II structure and function.

Due to the fact that corn smut continues to cause huge grain yield losses [63,64,65], scientists are increasingly undertaking research related to the genetic background determining resistance to this disease [66]. In the studies conducted by Zou et al. [64], it was shown that the phytohormone methyljasmonate (MeJA) can induce plant defense against microbial pathogens including *Ustilago maydis*. Other authors [67] consider salicylic acid (SA) and jasmonic acid (JA) to be important defense hormones. Fungal pathogens can activate defense responses associated with JA. Moreover, as a plant hormone, SA can interact with various plant hormone-related signaling pathways to activate the immune response and disease resistance of plants [68]. Therefore, there is a high probability that the gene encoding lutein deficient 5, chloroplastic selected by the team is involved in the immune response to stress induced by *Ustilago maydis* because, like salicylic acid and jasmonic acid, it has a strong antioxidant effect.

The association mapping used in this study has proven to be a promising approach compared to traditional mapping. It enabled the identification of candidate genes associated with maize resistance to smut. The literature reports discussed above confirmed that three of these genes (ATAD3, EDM2, and CYP97A3) could be involved in the resistance response of maize to smut infection. To date, two main types of association mapping have been characterized: genome-wide association mapping (GWAM) and candidate gene association mapping (CGAM). The GWAM approach analyzes genetic variability across the entire genome to identify association signals for various complex traits, while CGAM correlates DNA polymorphisms in selected candidate genes with the trait of interest [69,70]. There are many examples of successful applications of association analysis in cereals, particularly in maize. Recently, genome-wide association mapping (GWAM) has become a powerful tool for analyzing the genetic architecture of complex traits in various crop species [71]. Initially, association mapping performed in maize [72] did not consider population structure. This error was rectified by Pritchard, who included population structure in his study on maize in 2001 [73].

The current study demonstrated the utility of field, molecular, bioinformatics, and statistical analyses for identifying candidate genes associated with maize smut resistance. Moreover, methods for identifying candidate genes that could be used for selecting genotypes with desirable traits were proposed. This approach will enable cost savings compared to traditional methods of developing maize varieties.

## 4. Materials and Methods

### 4.1. Materials

The plant material consisted of 122 maize hybrids obtained from Hodowla Roślin Smolice sp. z o.o. (Smolice, Poland) IHAR Group (51°42′12″ N 17°10′10″ E) and Małopolska Hodowla Roślin sp. z o.o. (Kobierzyce, Poland) (50°58′17″ N 16°55′50″ E). Individual hybrids were created as a result of crossing inbred lines of different origins (Table 1).

### 4.2. Methods

**Figure 7:**
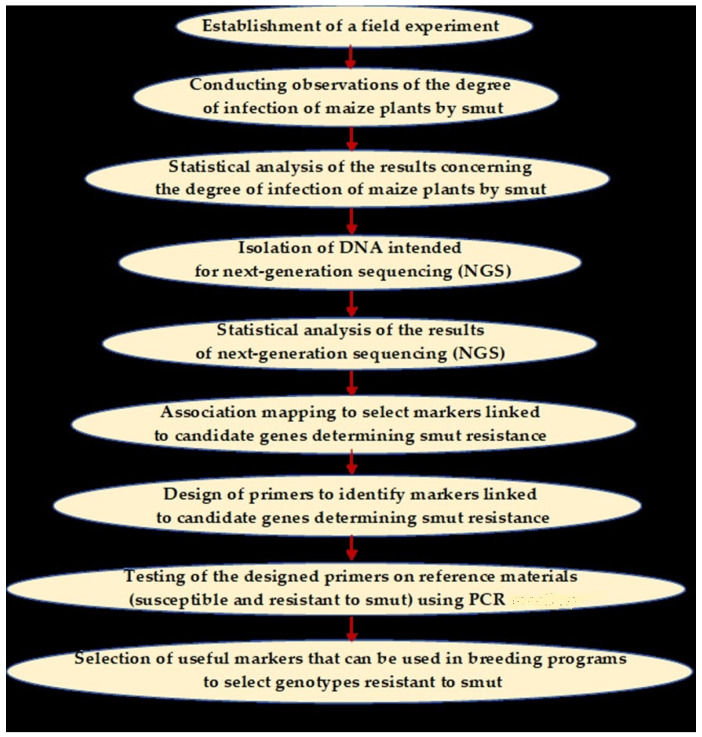
A diagram showing the subsequent stages of conducting the experiment.

Below is a flow chart showing the order of research conducted (Figure 7).

#### 4.2.1. Field Experiment

This experiment was established in plots of 10 m^2^, in three repetitions, in a randomized complete block design, at two locations: Smolice (51°42′58.904″ N, 17°13′29.13″ E) and Kobierzyce (50°58′19.411″ N, 16°55′47.323″ E). A health evaluation was carried out on ten plants from each plot by calculating the percentage of maize smut caused by the fungus *Ustilago maydis*.

#### 4.2.2. Weather Conditions

In Smolice (51°42′58.904″ N, 17°13′29.13″ E), the precipitation and air temperature in 2022 were unfavorable in the initial maize growing period. The average temperature in this year was 9.54 °C and was 0.8 °C higher than the fifty-year average. Low temperature and ground frosts in April and May caused, despite the early sowing date, maize to remain in the 2nd–3rd leaf stage for a long time, and purple discoloration appeared due to difficulties in absorbing phosphorus from soil. The warmest month in this area was August (20 °C), and the lowest temperature was recorded in December (−1.1 °C). The average precipitation in Smolice was 34.45 mm and was 13.82 mm lower than the long-term average. The most rainfall was recorded in July (55 mm) and the least in March (15 mm). The rainfall that occurred at the end of May had a positive impact on the development of maize. At the same time, in Kobierzyce (50°58′19.411″ N, 16°55′47.323″ E), a higher average air temperature was recorded compared to that in Smolice, i.e., 11.46 °C. This temperature was higher than the long-term average by 2.58 °C. In this area, the warmest month was August (22 °C), while the coldest was December (1.5 °C). Observing morphological features, there was no purple discoloration on the plants, which appeared on the plots in Smolice. In Kobierzyce, the average rainfall was 51.52 mm, which was 3.22 mm higher than the average long-term rainfall. The most rainfall was recorded in May—95.8 mm, and the least in March—21.6 mm.

#### 4.2.3. Extent of Maize Smut Infection

The globally applied BBCH scale (Biologische Bundesantalt, Bundessortenamt and Chemische Industrie, Erfurt, Germany) was used to determine the developmental stages of maize. The first field observations were made when maize was in the third leaf stage (BBCH13). At this time, plants are prone to dieback. This period spans from the end of May to the end of June. Infested plants grow poorly, are deformed, often produce side shoots, do not set ears, and can also die. Subsequent observations were carried out in July, during the heading and flowering stages (BBCH59-BBCH67). During this, period, the pathogen severely damages panicles and young ears, often due to hail or pest activity. If the fungus develops in the middle or upper part of the stalks and infect the ears, maize will fail to produce grain. The third stage of observation was conducted during the grain filling and milk maturity phases (BBCH71-BBCH75). During this period, smut infection does not significantly impact the yield, but it causes a deterioration of its quality (https://www.openagrar.de/servlets/MCRFileNodeServlet/openagrar_derivate_00010429/BBCH-Skala_de.pdf) accessed on 20 May 2024.

#### 4.2.4. DNA Isolation

DNA from 122 maize hybrids was isolated using the Plant DNA MINI Kit reagents from Syngen, Wrocław, Poland (SY261010). The concentration and purity of the isolated DNA samples were determined using a DS-11 spectrophotometer from DeNovix, Wilmington, DE, USA. The DNA template was diluted with deionized distilled water to achieve a uniform concentration of 100 ng μL^−1^. The prepared DNA samples were then subjected to next-generation sequencing (NGS).

#### 4.2.5. Genotyping

The isolated DNA from 122 maize hybrids (50 μL at 100 ng/μL) was transferred to two 96-well Eppendorf plates for next-generation sequencing. DArTseq technology, developed at Diversity Arrays Technology at the University of Canberra in Australia, was used for genotyping. Detailed methods can be found on the Diversity Arrays Technology website: https://www.diversityarrays.com/technology-and-resources/dartseq/, accessed on 15 April 2024.

#### 4.2.6. Association Mapping Using GWAS Analysis

Genotypic and phenotypic data were used to conduct association mapping to determine the relationships among the 122 analyzed maize hybrids. Mapping was performed using GWAS analysis. For the association analysis, only SilicoDArT and SNP sequences meeting the following criteria were selected: one SilicoDArT and/or SNP per sequence (69 nt), minor allele frequency (MAF) > 0.25, and missing observation fractions < 10%. Genotypic data were obtained from DArTseq analyses, while phenotypic data consisted of field observations on the degree of smut infection in maize plants caused by the fungus *Ustilago maydis*. SilicoDArT and SNP markers with the highest significance levels, i.e., those most strongly associated with plant resistance to maize smut, were selected for further research.

#### 4.2.7. Statistical Analysis and Association Mapping

Data were analyzed using an analysis of variance in a model with fixed effects of location and random effects of genotypes and genotype × location interaction. Association mapping, based on SilicoDArT and SNP data and average trait values, was conducted separately for data from the two locations using a mixed linear model method. The population structure was estimated by eigenanalysis and modeled using random effects [33,34]. All analyses and visualizations of the results were performed in GenStat 23.1, using the QSASSOCIATION procedure (https://genstat.kb.vsni.co.uk/wp-content/uploads/sites/10/StatsGuide.pdf) accessed on 28 May 2024. QSASSOCIATION is based on a mixed model marker–trait association analysis, also known as linkage disequilibrium mapping, with data from a single-environment trial. The control of genetic relatedness is necessary to avoid false positives in association mapping studies. The model was specified using the RELATIONSHIPMODEL eigenanalysis option, which infers the underlying genetic substructure in the population by retaining the most significant principal components from the molecular marker matrix. The scores of the significant axes are used as covariables in the mixed model, which effectively approximates the structuring of the genetic variance–covariance matrix by a coefficient of the coancestry matrix (kinship matrix). The significance of the association between maize smut and SilicoDArT and SNP markers was assessed using *p*-values corrected for multiple testing by the Benjamini–Hochberg method. Benjamini–Hochberg correction is a statistical method used for multiple comparisons to adjust significance levels and control the type I error rate (false positives). It is a method of conceptualizing the rate of type I errors in null hypothesis testing when conducting multiple comparisons. The false discovery rate (FDR) controlling procedures were designed to control the FDR, which is the expected proportion of “discoveries” (rejected null hypotheses) that are false (incorrect rejections of the null).

#### 4.2.8. Physical Mapping

SilicoDArT and SNP marker sequences selected in GWAS analysis were further examined using the Basic Local Alignment Search Tool (BLAST) and QIAGEN CLC Genomics Workbench software 24.0.2. The sequences were mapped to the complete maize reference genome sequence (Genome assembly B73 RefGen_v4). These analyses allowed for the determination of the chromosomal locations of the marker sequences. Subsequently, all gene sequences located in the identified chromosomal regions were subjected to further analysis.

#### 4.2.9. Functional Analysis of Gene Sequences

The functional analysis was performed using the Blast2GO 6.0 program, focusing on the sequences of all genes located in the designated chromosomal regions. The aim was to obtain information about the biological function of gene sequences located within these regions. Gene functional profiling utilized information from the Gene Ontology (GO, https://geneontology.org/, accessed on 15 April 2024) and Kyoto Encyclopedia of Genes and Genomes (KEGG, https://www.genome.jp/kegg/, accessed on 15 April 2024) databases.

#### 4.2.10. Primer Design for SilicoDArT and SNPs Linked to Maize Smut Resistance and Polymerase Chain Reaction (PCR)

PCR primers were designed using Primer 3 plus. The proposed settings were used to design the primers. Standard PCRs were conducted using a C1000 thermal cycler (Biorad, Hercules, CA, USA). Each reaction mixture contained the following components: 1 µ DNA template (50 ng µL^−1^), 4 µL polymerase buffer (5×), 1.6 µL dNTP (10 mM), 1.6 µL MgCl_2_ (25 mM), 0.5 µL forward primer (10 µM), 0.5 µL reverse primer (10 µM), 0.2 µL GoTaq polymerase (5 U µL^−1^), and 10.6 µL H_2_O. The composition was adjusted depending on the identified marker, and the PCR conditions were tailored for each marker, specifically varying the primer annealing temperature based on their melting temperatures. The amplification temperature profile used was as follows: initial denaturation for 5 min at 95 °C, followed by 35 cycles (denaturation for 45 s at 95 °C); primer annealing for 1 min at a temperature specific to each primer pair, consistent with their melting temperature; extension for 1 min at 72 °C; and a final elongation for 5 min at 72 °C followed by cooling to 4 °C.

#### 4.2.11. Electrophoresis

The electrophoresis of the PCR products was performed on a 2.5% agarose gel, with the addition of 1 µL of Midori Green solution, for 2 h at 100 V. O’RangeRuler 100 bp (Fermentas, Waltham, MA, USA) was used as a molecular weight marker to determine the sizes of the amplified products. The separated DNA fragments were visualized under UV light and captured as digital images using the BIORAD gel visualization and documentation system.

## 5. Conclusions

Current achievements in the field of plant biotechnology exceed previous expectations, and the prospects for their use are even more promising. Moreover, a better understanding of plant biology enabled by the use of “omics” technologies, exploiting molecular biology resources and new data analysis platforms, has been translated into agricultural practice and has enabled the improvement of many crop species, including maize. By using next-generation sequencing (NGS) and statistical tools, 10 statistically significant molecular markers associated with the resistance of maize plants to smut were identified in two locations (Smolice and Kobierzyce). Of the 10 selected markers, 3 SilicoDArT (24016548, 2504588, 4578578) and 3 SNP (4779579, 2467511, 4584208) markers were located within genes. According to literature reports, of these six genes, three (ATAD3, EDM2, and CYP97A3) are characterized proteins that may play a role in the immune response that develops in response to corn smut infection. In order to identify markers and their associated candidate genes, we designed primers for their identification. The identified molecular markers can be used for genotypic selection (MAS—marker-assisted selection), which can significantly support phenotypic selection and improve the breeding cycle. The wide implementation of MAS leads to the shortening of the breeding cycle, the length of which, according to breeders, is the main factor limiting breeding progress. Moreover, the identification and analysis of the function of new genes associated with maize resistance to smut will contribute to a better understanding of the maize defense mechanism against fungal diseases.

## Figures and Tables

**Table 1:** The origin of the analyzed genotypes.

	Parent Lines with Dent-Type Kernels		Parent Lines with Flint-Type Kernels	
Lp	Origins	Obtained Hybrids	Origin	Obtained Hybrids
1	Group related to the F2 line bred at INRA in France from the Lacaune population	G01.01, G01.02, G01.03, G01.04, G01.05, G01.06, G01.07, G01.08, G01.09, G01.10, G01.11, G01.12, G01.13, G01.14, G01.15, G01.16, G01.17, G01.18, G01.19, G01.20, G01.21	Group from the USA—Iowa Stiff Stalk Synthetic (BSSS)	G04.01, G04.02, G04.03, G04.04, G04.05, G04.06, G04.07, G04.08, G04.09, G04.10, G04.11, G04.12, G04.13, G04.14, G04.15, G04.16, G04.17, G04.18, G04.19, G04.20, G04.21
2	Group related to the EP1 line, bred in Spain from a population from the Pyrenees	G02.01, G02.02, G02.03, G02.04, G02.05, G02.06, G02.07, G02.08, G02.09, G02.10, G02.11, G02.12, G02.13, G02.14, G02.15, G02.16, G02.17, G02.18, G02.19, G02.20, G02.21	Group from the USA—Iowa Dent (ID)	G05.01, G05.02, G05.03, G05.04, G05.05, G05.06, G05.07, G05.08, G05.09, G05.10, G05.11, G05.12, G05.13, G05.14, G05.15, G05.16, G05.17, G05.18, G05.19, G05.20, G05.21
3	German Flint group	G03.01, G03.02, G03.03, G03.04, G03.05, G03.06, G03.07, G03.08, G03.09, G03.10, G03.11, G03.12, G03.13, G03.14, G03.15, G03.16, G03.17, G03.18, G03.19, G03.20, G03.21	Group from the USA—Lancaster	G06.01, G06.02, G06.03, G06.04, G06.05, G06.06, G06.07, G06.08, G06.09, G06.10, G06.11, G06.12, G06.13, G06.14, G06.15, G06.16, G06.17

**Table 2:** Statistical summary of 122 maize hybrid phenotypes affected by maize smut.

Location	Repeat	Mean	Skewness	Kurtosis	Coefficient of Variation (%)	Broad-Sense Heritability
Kobierzyce	Repeat 1	1.909 ± 3.084	1.895	3.353	61.60	59.7
	Repeat 2	2.673 ± 3.809	1.684	2.517	42.50	
	Repeat 3	2.449 ± 3.191	1.513	2.289	30.30	
	Mean	2.344 ± 3.383	1.734	2.924	44.33	
Smolice	Repeat 1	1.175 ± 2.278	2.299	4.999	93.87	
	Repeat 2	0.725 ± 1.554	2.589	7.552	88.34	
	Repeat 3	1.206 ± 2.031	2.104	4.501	68.41	
	Mean	1.035 ± 1.984	2.393	5.988	91.69	

**Table 3:** Two-way analysis of variance for maize smut.

Source of Variation	Degrees of Freedom	Sum of Squares	Mean Square	F Statistic
Location	1	313.26	313.26	57.09 ***
Genotype	121	2080.03	17.19	3.13 ***
Location × Genotype	121	856.27	7.08	1.29 *
Residual	488	2677.60	5.45	
Total	731	5927.16		

* *p* < 0.05; *** *p* < 0.001.

**Table 4:** The mean values and standard deviation (s.d.) of maize smut for individual genotypes in two locations and the average of the locations.

Location	Kobierzyce		Smolice		Average		Location	Kobierzyce		Smolice		Average	
Genotype	Mean	s.d.	Mean	s.d.	Mean	s.d.	Genotype	Mean	s.d.	Mean	s.d.	Mean	s.d.
G01.01	0	0	1.773	0.068	0.887	0.972	G03.20	0	0	0	0	0	0
G01.02	3.417	1.484	0.597	1.033	2.007	1.922	G03.21	3.377	1.518	1.817	3.147	2.597	2.369
G01.03	0	0	0.607	1.051	0.303	0.743	G04.01	1.71	1.482	0	0	0.855	1.325
G01.04	0.833	1.443	1.85	1.85	1.342	1.585	G04.02	3.333	1.443	0	0	1.667	2.041
G01.05	1.667	1.443	1.15	1.992	1.408	1.581	G04.03	8.333	8.036	3.703	4.9	6.018	6.471
G01.06	0.833	1.443	0	0	0.417	1.021	G04.04	2.5	2.5	0	0	1.25	2.092
G01.07	1.687	1.461	0.583	1.01	1.135	1.276	G04.05	1.667	1.443	0	0	0.833	1.291
G01.08	5.773	5.176	0	0	2.887	4.552	G04.06	3.42	5.924	0	0	1.71	4.189
G01.09	1.667	1.443	0	0	0.833	1.291	G04.07	1.667	2.887	0	0	0.833	2.041
G01.10	0	0	0	0	0	0	G04.08	3.42	1.595	0.617	1.068	2.018	1.957
G01.11	1.71	2.962	0.617	1.068	1.163	2.079	G04.09	4.523	4.305	1.147	0.993	2.835	3.351
G01.12	3.42	1.593	0.607	1.051	2.013	1.957	G04.10	0.853	1.478	0.573	0.993	0.713	1.137
G01.13	1.78	1.545	0	0	0.89	1.381	G04.11	5.02	2.47	1.17	2.026	3.095	2.921
G01.14	5.983	6.452	3.35	4.398	4.667	5.145	G04.12	0.833	1.443	0	0	0.417	1.021
G01.15	5.087	0.075	4.357	3.873	4.722	2.482	G04.13	3.333	1.443	0.667	1.155	2	1.871
G01.16	2.563	2.565	3.54	1.735	3.052	2.03	G04.14	1.667	2.887	1.17	2.026	1.418	2.247
G01.17	1.667	2.887	0.667	1.155	1.167	2.041	G04.15	0.833	1.443	1.723	2.985	1.278	2.153
G01.18	5.963	3.006	0.723	1.253	3.343	3.533	G04.16	0.833	1.443	0.597	1.033	0.715	1.13
G01.19	0	0	0	0	0	0	G04.17	0	0	1.17	2.026	0.585	1.433
G01.20	0.833	1.443	0	0	0.417	1.021	G04.18	0	0	0	0	0	0
G01.21	1.667	2.887	0	0	0.833	2.041	G04.19	0.833	1.443	0.63	1.091	0.732	1.15
G02.01	0	0	0	0	0	0	G04.20	1.667	2.887	6.173	2.142	3.92	3.356
G02.02	0.833	1.443	0	0	0.417	1.021	G04.21	7.563	6.567	0	0	3.782	5.866
G02.03	0	0	0.607	1.051	0.303	0.743	G05.01	3.333	2.887	0.573	0.993	1.953	2.452
G02.04	1.687	1.461	0	0	0.843	1.307	G05.02	0	0	1.17	1.014	0.585	0.907
G02.05	8.51	7.701	1.19	2.061	4.85	6.442	G05.03	0	0	3.49	3.51	1.745	2.93
G02.06	2.5	4.33	2.977	2.064	2.738	3.045	G05.04	1.627	2.817	0.583	1.01	1.105	1.977
G02.07	6.607	6.28	1.283	2.223	3.945	5.124	G05.05	0.853	1.478	0	0	0.427	1.045
G02.08	0.853	1.478	0.63	1.091	0.742	1.168	G05.06	0	0	0.583	1.01	0.292	0.714
G02.09	0.877	1.518	0	0	0.438	1.074	G05.07	0	0	0	0	0	0
G02.10	0.833	1.443	0	0	0.417	1.021	G05.08	0	0	0	0	0	0
G02.11	4.233	5.173	1.19	1.033	2.712	3.73	G05.09	0	0	0.64	1.109	0.32	0.784
G02.12	0	0	0	0	0	0	G05.10	0	0	0	0	0	0
G02.13	0	0	1.16	1.006	0.58	0.899	G05.11	1.667	2.887	0.563	0.976	1.115	2.02
G02.14	5.97	3.781	4.977	1.26	5.473	2.579	G05.12	8	3.056	2.87	1.992	5.435	3.636
G02.15	9.34	5.092	7.273	4.81	8.307	4.572	G05.13	0.833	1.443	0.617	1.068	0.725	1.142
G02.16	0.877	1.518	1.853	3.21	1.365	2.309	G05.14	9.167	1.443	5.017	3.837	7.092	3.448
G02.17	0.833	1.443	0	0	0.417	1.021	G05.15	0	0	0	0	0	0
G02.18	3.377	3.805	0	0	1.688	3.035	G05.16	0.833	1.443	0.583	1.01	0.708	1.123
G02.19	0.833	1.443	1.733	1.755	1.283	1.52	G05.17	1.78	1.545	0	0	0.89	1.381
G02.20	3.42	2.965	0	0	1.71	2.65	G05.18	0.877	1.518	1.237	1.076	1.057	1.193
G02.21	0.833	1.443	1.797	1.785	1.315	1.545	G05.19	0	0	0	0	0	0
G03.01	5	5	2.45	2.838	3.725	3.895	G05.20	0	0	0.53	0.918	0.265	0.649
G03.02	5.753	5.583	0.757	1.311	3.255	4.544	G05.21	1.71	2.962	0.64	1.109	1.175	2.084
G03.03	4.21	3.834	0	0	2.105	3.346	G06.01	0	0	0	0	0	0
G03.04	1.667	2.887	1.19	2.061	1.428	2.258	G06.02	0	0	0.583	1.01	0.292	0.714
G03.05	0	0	0	0	0	0	G06.03	0.833	1.443	0	0	0.417	1.021
G03.06	0	0	0.617	1.068	0.308	0.755	G06.04	1.71	2.962	1.817	3.147	1.763	2.734
G03.07	2.5	4.33	0	0	1.25	3.062	G06.05	5	5	0	0	2.5	4.183
G03.08	0.833	1.443	0	0	0.417	1.021	G06.06	1.667	2.887	1.723	2.985	1.695	2.626
G03.09	1.627	2.817	0	0	0.813	1.992	G06.07	4.25	2.979	0	0	2.125	2.995
G03.10	1.687	1.461	1.257	2.177	1.472	1.675	G06.08	8.333	5.204	0.653	1.132	4.493	5.389
G03.11	0	0	0	0	0	0	G06.09	3.49	3.894	0	0	1.745	3.118
G03.12	0	0	0.573	0.993	0.287	0.702	G06.10	2.5	0.06	0	0	1.25	1.37
G03.13	6.84	2.737	3.557	3.419	5.198	3.303	G06.11	4.167	5.204	2.383	2.065	3.275	3.673
G03.14	1.71	2.962	0	0	0.855	2.094	G06.12	1.687	1.461	3.593	0.04	2.64	1.395
G03.15	3.333	1.443	1.283	2.223	2.308	2.018	G06.13	2.52	2.5	2.483	2.893	2.502	2.418
G03.16	5	5	0.607	1.051	2.803	4.029	G06.14	0	0	0.583	1.01	0.292	0.714
G03.17	0.833	1.443	0.617	1.068	0.725	1.142	G06.15	1.687	1.461	1.797	1.82	1.742	1.478
G03.18	8.46	7.805	0.583	1.01	4.522	6.587	G06.16	4.107	3.741	3.573	4.725	3.84	3.823
G03.19	0	0	0	0	0	0	G06.17	7.5	2.5	6.293	3.477	6.897	2.788
Location	2.344	3.383	1.035	1.984									
LSD_0.05_—Genotype: 2.657; Location: 0.340; Genotype × Location: 3.757													

LSD—Least Significant Difference.

**Table 5:** SilicoDArT and SNP molecular markers significantly associated with maize plant resistance to smut in Smolice and Kobierzyce (significant associations selected at *p* < 0.05 with Benjamini–Hochberg correction for multiple testing).

Type of Markers		All	SilicoDArT	SNP
Smolice				
Number of markers		2609	2136	473
Negative	Number	1822	1552	270
	Effects	−6.29–−0.52	−6.29–−0.52	−6.29–−0.52
	Percentage variance accounted for	2.4–20.1	2.4–20.1	2.4–17.2
	LOD	1.30–6.87	1.30–6.87	1.30–5.91
Positive	Number	787	584	203
	Effects	0.52–1.68	0.52–1.68	0.52–1.60
	Percentage variance accounted for	2.4–15.4	2.4–15.4	2.4–13.3
	LOD	1.30–5.35	1.30–5.35	1.30–4.66
Kobierzyce				
Number of markers		7331	5775	1556
Negative	Number	4487	3654	833
	Effects	−7.05–−0.87	−7.05–−0.87	−7.05–−0.869
	Percentage variance accounted for	2.4–15.3	2.4–15.3	2.4–12.7
	LOD	1.30–5.31	1.30–5.31	1.30–4.79
Positive	Number	2844	2121	723
	Effects	0.87–2.56	0.87–2.56	0.87–2.17
	Percentage variance accounted for	2.4–12.0	2.4–11.9	2.4–12.0
	LOD	1.30–4.27	1.30–4.23	1.31–4.27

**Table 6:** Sixty-one statistically significant markers (LOD > 2.3) in both localities: Kobierzyce and Smolice.

Chr	Marker Type	CloneID	Kobierzyce			Smolice		
			Estimate	Percent ^1^	LOD	Estimate	Percent	LOD
1	SilicoDArT	4775057	−7.02	12.7	4.48	−5.20	20.1	6.87
1	SilicoDArT	16721763	−3.70	10.1	3.67	−2.12	9.3	3.42
1	SilicoDArT	4765073	1.39	7.3	2.82	0.85	7.8	2.97
1	SilicoDArT	5589311	−1.44	7.8	2.98	−0.86	7.8	2.97
1	SilicoDArT	9705615	1.66	9.4	3.44	0.90	7.6	2.89
1	SNP	4772326	1.44	6.8	2.65	0.89	7.4	2.85
1	SilicoDArT	4776097	−3.49	7.3	2.82	−1.88	5.8	2.37
2	SilicoDArT	2450125	−2.04	6.4	2.53	−1.64	12.2	4.34
2	SNP	4773308	−4.84	8.7	3.25	−3.20	10.9	3.93
2	SilicoDArT	60118158	−2.30	5.9	2.41	−1.43	6.6	2.60
3	SilicoDArT	5586961	−2.94	9.2	3.40	−1.83	10.1	3.68
3	SilicoDArT	4768941	−1.44	5.8	2.37	−1.06	9.4	3.46
3	SilicoDArT	25979976	−1.58	6.7	2.62	−1.01	7.7	2.95
3	SilicoDArT	2508842	2.03	8.8	3.27	1.13	7.6	2.92
3	SilicoDArT	24016422	−1.50	6.5	2.56	−0.86	6.0	2.41
4	SNP	4779579	−7.05	6.1	2.44	−6.29	14.6	5.09
4	SilicoDArT	24016584	−2.08	15.3	5.31	−1.01	9.9	3.60
4	SilicoDArT	24026851	−1.49	8.5	3.19	−0.95	9.8	3.58
4	SNP	4771165	1.35	6.9	2.69	0.89	8.6	3.22
4	SNP	4764698	−1.33	6.7	2.65	−0.88	8.4	3.17
4	SilicoDArT	24015366	−1.33	6.5	2.57	−0.87	8.0	3.05
4	SNP	2589540	−1.72	7.1	2.76	−1.07	7.8	2.96
4	SNP	4764301	−1.72	7.1	2.76	−1.07	7.8	2.96
4	SNP	4779191	1.31	6.5	2.57	0.85	7.7	2.95
4	SilicoDArT	2442822	−1.63	6.3	2.52	−1.05	7.5	2.89
4	SilicoDArT	58293207	1.24	5.7	2.34	0.83	7.5	2.88
4	SilicoDArT	9682733	1.56	9.5	3.49	0.83	7.4	2.84
4	SNP	4779077	1.26	6.0	2.41	0.82	7.3	2.82
4	SNP	5585252	−1.74	12.1	4.29	−0.81	7.0	2.72
4	SilicoDArT	4767508	1.89	8.5	3.18	1.03	6.9	2.69
4	SilicoDArT	5586088	−1.64	6.9	2.68	−0.98	6.9	2.68
4	SNP	4773799	−1.72	7.1	2.76	−1.00	6.8	2.67
4	SilicoDArT	4774125	1.45	8.1	3.07	0.77	6.3	2.53
4	SilicoDArT	7054837	1.26	5.9	2.39	0.77	6.3	2.52
4	SNP	4582733	1.26	5.9	2.39	0.77	6.3	2.52
4	SilicoDArT	2502010	−5.29	6.8	2.68	−3.03	6.3	2.51
4	SilicoDArT	7049831	1.60	6.4	2.55	0.93	6.2	2.48
4	SilicoDArT	2489736	1.42	7.7	2.95	0.76	6.1	2.45
4	SilicoDArT	4587524	−1.49	7.4	2.86	−0.80	5.9	2.39
4	SilicoDArT	2426908	1.43	7.8	2.96	0.74	5.6	2.31
5	SNP	2467511	−5.35	10.8	3.90	−3.95	17.2	5.91
5	SilicoDArT	2504588	−7.05	6.1	2.44	−6.29	14.6	5.09
5	SilicoDArT	4576740	1.29	6.2	2.48	0.92	9.3	3.41
5	SilicoDArT	2508818	−3.48	7.2	2.80	−2.12	7.7	2.93
5	SilicoDArT	2535150	1.66	8.2	3.09	0.92	7.1	2.75
5	SilicoDArT	2549538	1.42	7.7	2.93	0.78	6.5	2.56
6	SilicoDArT	25941876	−2.68	6.6	2.62	−1.98	10.7	3.84
7	SilicoDArT	2500468	2.00	9.2	3.41	1.42	13.5	4.72
7	SilicoDArT	2638595	1.93	8.8	3.29	1.28	11.3	4.04
7	SilicoDArT	29619676	1.24	5.6	2.31	0.95	9.8	3.57
7	SilicoDArT	4586304	1.68	7.2	2.79	1.14	9.6	3.51
7	SNP	2516640	1.68	7.2	2.79	1.14	9.6	3.51
7	SNP	2456458	1.90	7.6	2.91	1.16	8.0	3.03
7	SilicoDArT	25949617	−2.19	11.2	4.01	−1.10	7.8	2.98
7	SilicoDArT	67862016	−2.62	13.9	4.84	−1.19	7.8	2.96
8	SNP	4584208	−5.63	12.1	4.29	−3.14	10.5	3.80
8	SilicoDArT	9706071	−3.48	10.3	3.74	−2.04	10.0	3.64
8	SilicoDArT	4766929	−1.65	8.3	3.14	−0.85	6.1	2.45
9	SilicoDArT	4766696	−1.63	5.9	2.38	−1.19	9.2	3.40
10	SilicoDArT	4578678	−3.86	11.0	3.96	−2.98	19.1	6.55
10	SilicoDArT	4776674	−3.66	6.4	2.55	−2.26	6.9	2.71

^1^ Percentage variance accounted for.

**Table 7:** Characteristics and location of markers significantly associated with resistance to maize smut in both localities (Smolice and Kobierzyce).

Marker	Marker Type	Chromosome	Marker Location	Candidate Genes
4775057	SilicoDArT	Chr.1	77754855	LOC100278950 transcription initiation factor TFIID subunit 4b/NC_050096.1 (77603380…77617814) 135 Mbp downstream
24016584	SilicoDArT	Chr.4	165282490	LOC103653966 ATPase family AAA domain-containing protein 3C/NC_050099.1 (165277362…165282300) 186 bp downstream
4779579	SNP[F]10-27:G>A-27:G>A	Chr.4	204565922	LOC103654479 enhanced downy mildew 2 (EDM2)/NC_050099.1 (204537019…204567358)
2467511	SNP[F]10-59:G>A-59:G>A	Chr.5	224222016	LOC103627895 lutein deficient 5, chloroplastic/NC_050100.1 (224217814…224223263)
2504588	SilicoDArT	Chr.5	221680423	LOC103627849 uncharacterized ncRNA/NC_050100.1 (221679742…221681266)
2500468	SilicoDArT	Chr.7	166187027	LOC541728 transcription factor MYB39/NC_050102.1 (166182362…166184351)
67862016	SilicoDArT	Chr.7	6168374	LOC103633583 two-component response regulator-like PRR1/NC_050102.1 (6153359…6155010)
4584208	SNP[F]10-42:C>A-42:C>A	Chr.8	38660613	LOC100277329 uncharacterized protein/NC_050103.1 (38658283…38661948)
9706071	SilicoDArT	Chr.8	110928069	LOC100279375 uncharacterized protein/NC_050103.1 (110879661…110881883)
4578678	SilicoDArT	Chr.10	100807159	LOC100285431 uncharacterized protein/NC_050105.1 (100804448…110881883)

**Table 8:** Sequences of primers designed to identify newly selected markers significantly associated with maize smut resistance.

Marker	Primer Sequences		Tm °C	Size
	Forward	Reverse		
4775057	GTCATATGTGGGACCAAATCTGCAG	AGTACTCGAACTTGCACACGA	61	140
24016584	CTAATTGTCTACAATGTTACTGCAG	ACGTAGAGTTTACATGATTCGGG	59	127
4779579	AGCCAATTTGAGACATAAACTGCAG	GACTGGCATGAATACCATAGCG	60	559
2467511	CAATCATCGCAGTCACATACTGCAG	CCGACATATCTGCTTGTTGTGGT	62	98
2504588	TTTTTTCTTCTTCTCCTTGCTGCAG	CCTTCCTCGCCGATAGCTG	61	181
2500468	ACAGGTGTGCCACCTGCTGAT	TGCTGCACACGGAGAGACAC	64	143
67862016	TGACAGGTTAATAGGCTGCAG	ATTGAAACCTTTTGGCTAGTTGGT	59	79
4584208	AAAGTGACAGGTTAATAGGCTGCAG	GCGATGTTTCCACAGCCACC	62	125
9706071	CGGCGGTAGAGATGTAGGCCTGCAG	TATCGAGCCAGCCCAACATGGGA	67	269
4578678	TGCCATTAGTATGTTTGGGCTGCAG	ATAAACTACAACCACACTGGAGCTG	63	187

## Data Availability

The data presented in this study are available on request from the corresponding author. The data are not publicly available due to because the sequencing data is too large to be included in a supplement.
